# Objective Ventricle Segmentation in Brain CT with Ischemic Stroke Based on Anatomical Knowledge

**DOI:** 10.1155/2017/8690892

**Published:** 2017-02-07

**Authors:** Xiaohua Qian, Yuan Lin, Yue Zhao, Xinyan Yue, Bingheng Lu, Jing Wang

**Affiliations:** ^1^College of Electronic Science and Engineering, Jilin University, Changchun 130012, China; ^2^Division of Research and Innovations, Carestream Health, Inc., Rochester, NY 14615, USA; ^3^Affiliated Hospital of the Changchun University of Chinese Medicine, Changchun 130021, China; ^4^Collaborative Innovation Center of High-End Manufacturing Equipment, Xi'an Jiaotong University, Xi'an 710054, China

## Abstract

Ventricle segmentation is a challenging technique for the development of detection system of ischemic stroke in computed tomography (CT), as ischemic stroke regions are adjacent to the brain ventricle with similar intensity. To address this problem, we developed an objective segmentation system of brain ventricle in CT. The intensity distribution of the ventricle was estimated based on clustering technique, connectivity, and domain knowledge, and the initial ventricle segmentation results were then obtained. To exclude the stroke regions from initial segmentation, a combined segmentation strategy was proposed, which is composed of three different schemes: (1) the largest three-dimensional (3D) connected component was considered as the ventricular region; (2) the big stroke areas were removed by the image difference methods based on searching optimal threshold values; (3) the small stroke regions were excluded by the adaptive template algorithm. The proposed method was evaluated on 50 cases of patients with ischemic stroke. The mean Dice, sensitivity, specificity, and root mean squared error were 0.9447, 0.969, 0.998, and 0.219 mm, respectively. This system can offer a desirable performance. Therefore, the proposed system is expected to bring insights into clinic research and the development of detection system of ischemic stroke in CT.

## 1. Introduction

Computed tomography (CT) is generally used to assess patients with acute ischemic stroke in America, because of its faster speed, the better contrast of bones and blood, and the lower cost than magnetic resonance images (MRI). The ischemic stroke and cerebrospinal fluid (CSF) regions have a similar appearance in CT images; thus, accurate ventricle segmentation can significantly facilitate ischemic stroke region localization and is an indispensable step for the development of computer-aided detection (CAD) for acute ischemic stroke.

Several state-of-the-art methods have been proposed to segment ventricles in MRI [1], including active contour-based methods [2–4], fuzzy schemes [5, 6], and probability methods [7, 8]. However, these methods may be inappropriate to work on CT images, since there are lower contrast, higher noise level, and larger slice thickness in brain CT images.

Only little literature on the segmentation of brain CT images has been published. For example, Wei et al. proposed a segmentation scheme based on 2D Otsu thresholding approach [9]. Lee et al. applied the *k*-means and expectation maximization clustering to segment CT images [10]. Another method by Chen et al. was based on a Gaussian mixture model [11]. Gupta et al. integrated the adaptive threshold, connectivity, and domain knowledge to classify the cerebrospinal fluid, white matter, and gray matter on CT images [12]. These methods mentioned above were not designed specifically for ventricle segmentation and were not validated on the images with severe abnormalities. Chen et al. developed a ventricular segmentation system by combining low-level segmentation and high-level template matching [13]. Similarity, Liu et al. proposed a model-guided segmentation for ventricle region [14]. The two methods are both based on the template or model scheme for ventricle extraction in CT. Since these templates were yielded from the MRI brain image and registration was linear, the templates only provided a rough mask for the ventricle segmentation. Therefore, it is still challenging for these two methods to exclude stroke regions from segmentation results. Qian et al. proposed a level set model to segment CSF, but the result includes the stroke regions [15]. This study will improve the methods and extensively validate our previous work [16].

The significant difficulty of the accurate ventricle segmentation is to deal with CT images of patients with ischemic stroke. Some of the stroke regions and ventricles are connected and have similar intensities. To address this challenge, we developed an objective segmentation strategy of brain ventricles in unenhanced CT with ischemic stroke. We applied the following three schemes to exclude the stroke regions from segmentation results:

1. We took the largest three-dimensional (3D) connected component in a preliminary segmentation as the ventricular region, removing the lesion or other regions without the 3D connectivity relationship with the ventricle, since the initial segmentation result contained not only the ventricle but also some nonventricular regions, such as lesion or CSF.
2. The large stroke regions were removed by the image difference method. The large stroke areas tend to close the brain edge, and their intensities were generally lower than that of the main parts of ventricles. Thus, the stroke region can be extracted by the difference between segmentation results from two optimal threshold values.
3. The small stroke regions were removed by the adaptive template algorithm. The adaptive template was directly generated from the corresponding image itself based on the big intensity difference between the main part of the ventricle and the brain parenchyma. This template did not contain the whole ventricle but did cover the main part of the ventricular region. Thus, we applied this template to remove the small lesions around the main part of the ventricle, which was not subjected to the registration. Another effect was that the exclusion of these small lesions might break the connectivity relationship between the lesion regions and the ventricular region in 3D space.

## 2. Materials and Methods

**Figure 1:**
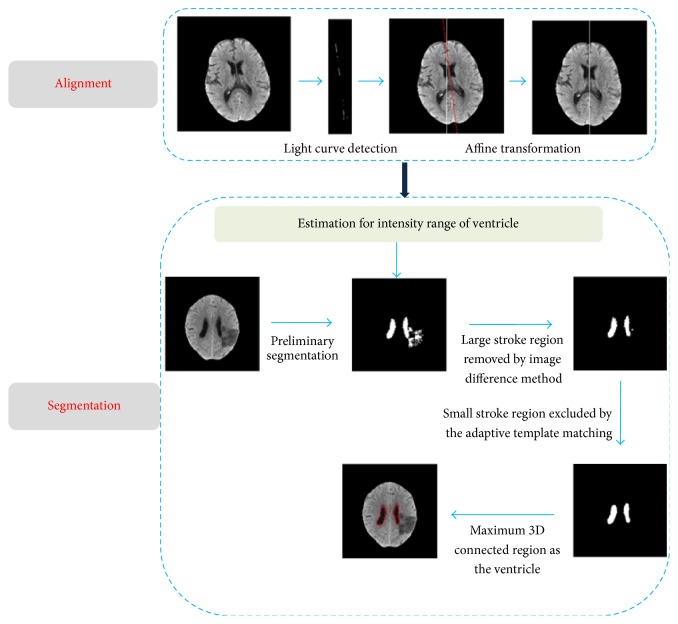
Schematic framework for segmentation of the brain ventricle in CT of patients with ischemic stroke.

As shown in Figure 1, the automated ventricle segmentation method is comprised of two phases, that is, alignment phase (Section 2.2) and segmentation phase (Section 2.3). In the alignment phase, the light curves/segment of the brain was detected to determine the midsagittal line for each slice. We then aligned the midsagittal line (MSL) with the vertical line of each slice to achieve brain alignment. In the segmentation phase, we first estimated the intensity range of the ventricle region based on clustering technique, connectivity, and domain knowledge. An image difference algorithm was developed to identify and remove the large stroke regions in the initial segmentation. The remaining small stroke region was further excluded by an adaptive template of the ventricle. Finally, the largest 3D connectivity of the segmented ventricle was employed to refine the segmentation result.

### 2.1. Dataset

We tested the proposed method on 50 CT scans of patients with ischemic stroke in this study. This dataset was collected from Jilin University Medical Center using CT scanners (Light Speed 16, GE Medical System) with an X-ray tube voltage of 120 kVp. Each patient has 14 slices with the thickness of 5 mm in this study. The matrix size of each slice is 512 × 512 pixels, and the pixel size is 0.426 mm with a 16-bit gray level. The 50 patients were composed of 29 males and 21 females, and their average age is 57 years with the range between 41 years and 76 years. We established a reference standard of ventricle for evaluation of segmentation result. A medical physicist (XQ, eight years of experience) manually delineated the ventricle boundaries for all the slices on an LCD screen as the reference standard to assess the accuracy of segmentation results.

### 2.2. Alignment of the Brain Image

Prior to the alignment of brain image, the skull was stripped by a threshold method since CT number of bone tissues are consistently higher than brain tissues. Generally, the CT number of soft tissue is less than 60 Hounsfield units (HU) (such as 1–12 HU of ventricle, 25–38 HU of white matter, and 35–60 HU of gray matter), while average CT intensity is 1000 HU for bones. Thus, we extracted the skull using a fixed threshold of 100 HU. The region inside the skull was considered as brain region and the region outside the skull served as background.

After the extraction of the brain, the inclination angle and position were corrected by aligning MSL with the vertical centerline of each slice. The determination of MSL is a key step in this alignment. Since the falx cerebri (i.e., narrow light curve/segment) presents on about 30% images, we applied the falx cerebri as a reference to identify the MSL. Therefore, we utilized two steps to achieve alignment of the brain, including (1) detection of a light curve in the brain and (2) affine transformation based on MSL.

#### 2.2.1. Detection of Light Curves in Brain

**Figure 2:**
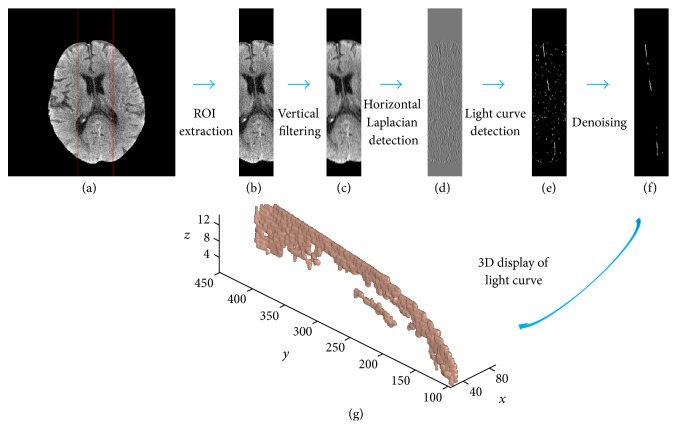
Diagram of light curve/segment detection: (a) original image without skull; (b) the ROI of the light curve; (c) the vertical filtered ROI; (d) the Laplacian image; (e) the detected light curve; (f) denoising light curve; (g) 3D display of light curves: *z*-axis represents the slice number; *x*- and *y*-axes denote the pixel number.

Figure 2 shows the schematic diagram of light curve detection. To accelerate the detection, we defined a rectangle region of interest (ROI), whose size was chosen to include the light curves to be detected. We selected a smallest minimum bounding rectangle of the brain area in the whole scan and then defined the half width of this rectangle as the width of the ROI. The height of the ROI was taken the default value of 512. Figure 2(b) shows the rectangle ROI of the brain.

CT brain image has a high level of noise. The common filtering may blur the weak edge, making detection of the light curve difficult. The light curve has a slight angle with the vertical direction; however, it is still regarded as vertical. Thus, we designed a one-dimensional (7 × 1) Gaussian filter with the variance of 2 to smooth the image along the vertical direction, which can preserve the edge information of the light curve in the horizontal direction as shown in Figure 2(c).

We then design a horizontal Laplacian detection mask, that is, [0.5,0, 1,0, 0.5], to detect the light curve, since the vertical strip included more edge points of the light curve than other places. With the Laplacian image (Figure 2(c)), we employed an adaptive threshold to yield an edge map, as shown in Figure 2(d). We empirically set the threshold as the average value with 2.5 multiple of the standard deviation of the Laplacian image.

After that, we erased the small unconnected noise point clouds in the edge map based on 3D connectivity. The noise points in edge map may negatively affect the subsequent 3D fitting of the middle sagittal plane. However, the 3D connected volume of these noise points is small; thus, we can remove them with a threshold in 3D connected volume. In our experiment, we applied thirty pixels as the threshold to obtain the clean edge map of light curve (Figure 2(f)). Figure 2(g) shows the 3D edge map of light curves.

#### 2.2.2. Affine Transformation Based on MSL

To obtain the precise MSL, we first fitted a middle sagittal plane in 3D Euclidean space through a set of edge segments of light curves using least-squares fitting approach. Let (*x*_*i*_, *y*_*i*_, *z*_*i*_) be a point of edge segments, which has totally *M* points and *i* = 1, 2,…, *M*. So, the optimum fitting plane can be achieved by the following formulation as

(1) $$\begin{array}{c} \left(\;a^{*},b^{*},c^{*}\;\right)=\underset{\left(a,b,c\right)}{\arg\min}\overset{M}{\underset{i=1}{\sum }}{\left(\;z_{i}-ax_{i}-by_{i}+c\;\right)}^{2}. \end{array}$$

The MSL of each slice was defined as the intersection line between the image and middle sagittal plane. Let *z*_*i*_ denote the *i*th slice of 3D image, and we can obtain the MSL of this slice as

(2) $$\begin{array}{c} ax-by=z_{i}-c. \end{array}$$

**Figure 3:**
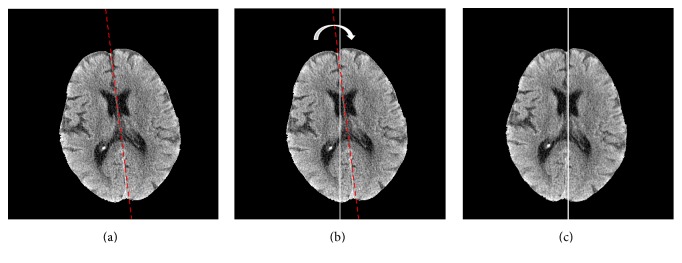
Alignment of the brain image: (a) original image with the midsagittal line (MSL, dashed line); (b) the vertical center line of a slice with white color and the MSL; (c) aligned brain image.

The determined MSL was shown in Figure 3(a). Finally, we aligned the MSL of the brain with the vertical center line of a slice using the affine transformation defined by

(3) x′=x−x0cos⁡θ+y−y0sin⁡θ+x0y′=x−x0sin⁡θ+y−y0cos⁡θ+y0,

where (*x*_0_, *y*_0_) is the center point of the vertical center line of a slice and *θ* is the inclination angle between the MSL and vertical center line. Figures 3(b) and 3(c) show that the inclination angle and position of the brain were corrected.

### 2.3. Segmentation of the Ventricle

**Figure 4:**
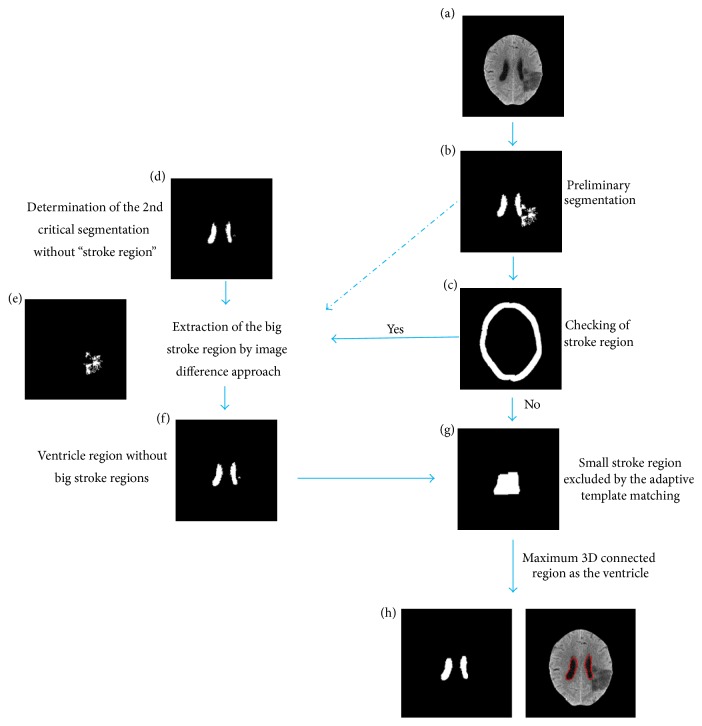
Flowchart of the exclusion of stroke area in the ventricular segmentation result.

In the phase of ventricle segmentation, we focused on excluding the stroke area in the ventricle segmentation result. The flowchart was shown in Figure 4.

#### 2.3.1. Parameter Estimation for the Ventricle

Prior to the segmentation of ventricle, we estimated parameters of the intensity distribution of the ventricle. We first applied the *K*-means algorithm (*K* = 2) on the 3D images for stratification of the brain image and took the largest 3D connected component of low-intensity category as the ventricle. Then, an estimation method based on connectivity and domain knowledge from the literature [8] was utilized to compute the intensity distribution of different tissues. Specifically, we tracked the slop of the histogram corresponding to the 3D largest connected component in rough intensity range of ventricle to determine a critical intensity, which serves as an initial classifier of cerebral spinal fluid and white matter. Thresholds of cerebral spinal fluid, white matter, and gray matter are optimally derived to minimize spatial overlap errors in different tissue types. In this study, ventricular intensity range of $\left[\begin{array}{cc} V_{min} & V_{max} \end{array}\right]$ will be adopted to extract the ventricular region.

#### 2.3.2. Preliminary Segmentation for the Ventricle Based on Estimated Parameters

*V*_max_, the estimated maximum of ventricular intensity range, was applied as a threshold value for preliminary segmentation of the ventricle. If the intensity range of the stroke is greater than *V*_max_, the preliminary segmentation is a good result. Whereas, if the intensity range of the stroke is less than *V*_max_, the segmentation result may be unacceptable, since it may also contain some stroke regions.

Then, we utilized the 3D connectivity of the preliminary segmentation result to obtain the largest volume as the initial segmentation of the ventricle. The stroke regions or noise areas without the 3D connectivity to the ventricle could be excluded by this step. Figure 4(b) shows that the large stroke regions are connected to the ventricle in the segmentation.

#### 2.3.3. Detection of the Big Stroke Regions

Since big stroke regions are mainly related to the anterior cerebral artery or middle cerebral artery, these stroke regions are mostly closed to the brain edge. Thus, we proposed a brain edge checking algorithm to determine whether the big stroke regions exist in the segmentation result. An annular region of the brain edge was defined to detect the objects. Assumed that the minimum side length of the minimum bounding rectangle of the brain was *L*_min_, the width of the annular region could be calculated by 0.15 × *L*_min_ to avoid some parts of the ventricle falling within the annular region. The mask of the brain edge annular region was shown in Figure 4(c). Thus, if the objective area was greater than the threshold, we labeled it as the stroke region. The threshold was empirically selected as 20 pixels to allow the presence of noise.

#### 2.3.4. Determination of the Big Stroke Regions

We proposed an image difference technique based on the heuristic searching algorithm to extract the big stroke regions, which were successfully detected in the preliminary segmentation by the edge checking method. This image difference technique essentially applied the difference between two segmentation results by different threshold values for determining the stroke regions. We first defined the critical threshold value (i.e., *T*_critical_). If a threshold was greater than *T*_critical_, the stroke regions in the segmentation result of this threshold could be detected by the edge analysis method; whereas, if the threshold was smaller or equal to *T*_critical_, none stroke region could be detected. We then obtained the stroke regions by

(4) $$\begin{array}{c} \text{PA}\approx f\left(\;V_{max}\;\right)-g\left(\;f\left(\;T_{\text{critical}}\;\right)\;\right), \end{array}$$

where *f*(*∗*) was the threshold method; *g*(*∗*) represented the subsequent refine algorithms, such as morphology method; and PA represented the stroke regions. So, we obtained the ventricle areas:

(5) $$\begin{array}{c} f\left(\;V_{max}\;\right)-g\left(\;\text{PA}\;\right). \end{array}$$

The vital step in the image difference method is to determine the critical threshold value *T*_critical_. We applied the gold searching method and the edge checking method to obtain the *T*_critical_ in range $\left[\begin{array}{cc} V_{min} & V_{max} \end{array}\right]$.

#### 2.3.5. Exclusion of the Small Stroke Regions

Some small stroke regions may still present in the segmentation result from the image difference approach. To address this problem, we developed an adaptive template matching approach, which applied the mask of the main part of the ventricle to exclude the remaining small stroke regions. The template was generated from each image. It did not contain the whole ventricle but covers the main part of the ventricle.

**Figure 5:**
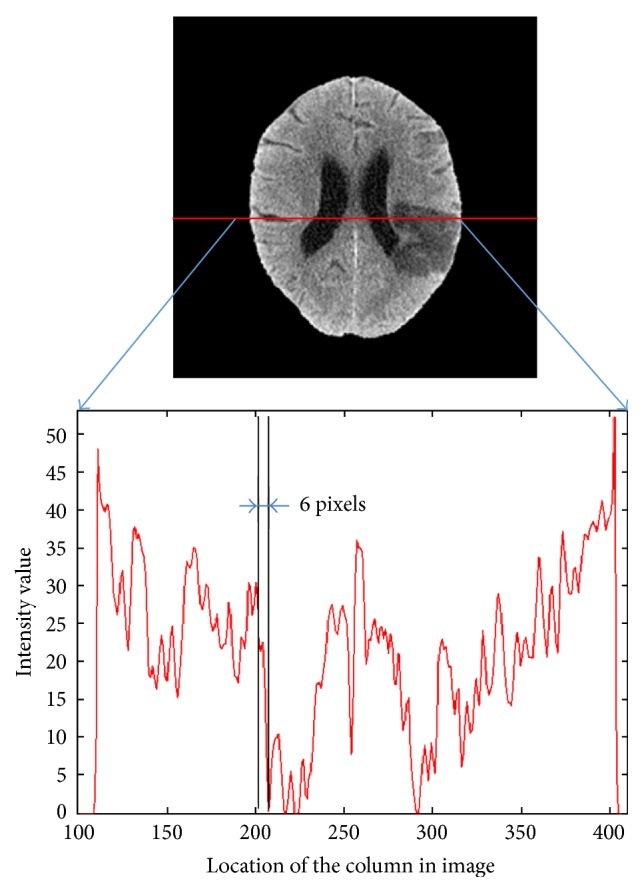
A sectional view of the gray-scale map for brain image.

**Figure 6:**
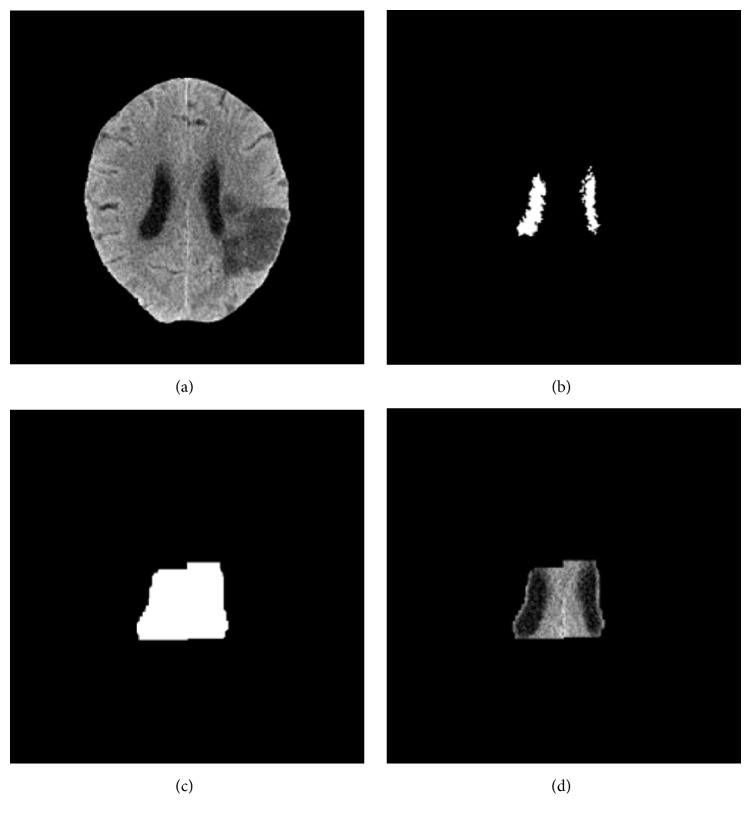
Generation of the template for ventricle: (a) original image; (b) initial segmentation result; (c) the generated template; (d) the corresponding brain area in the template.

Figure 5 shows a sectional view of the gray-scale map for a brain image. The intensity difference between the ventricle and brain parenchyma was around 20 intensity values, while the transition area was only 6 to 7 pixels. Thus, we applied *V*_min_ as a threshold for ventricle segmentation and took the 3D largest connected region as the ventricle, as shown in Figure 6(b). The ventricle segmentation, merely containing the right and left lateral ventricles and without the 3rd and 4th ventricle, was adaptively selected as the templates. To ensure that the template covers the ventricle, we conducted some morphological analysis, including closed operation and expansion operation. The generated template was shown in Figures 6(c) and 6(d).

After these steps, we linearly registered the template with the corresponding segmentation. The objects within the template served as the ventricle so that the remaining small stroke areas could be excluded from the segmentation results.

#### 2.3.6. Refinement of the Ventricular Segmentation

We employed connected component labeling to the segmented ventricle region. The largest volume served as the ventricular. We then removed the calcification regions in the results and smoothed the ventricular edges using the morphologically closed operation.

### 2.4. Evaluation of the Segmentation Method

We applied four measures, including Dice metric (Dice), root mean squared error (RMSE), reliability (*ℛ*)^28^, and correlation coefficient (*R*), to assess the performance of the proposed segmentation method. The four measures are defined as follows.

*(1) Dice Metric.* Let *V*_*s*_ represent the automatically segmented volume and *V*_*r*_ represent the manual segmentation (i.e., reference standard). The Dice is defined as

(6) $$\begin{array}{c} \text{Dice}=\frac{2V_{s}\cap V_{r}}{V_{s}+V_{r}}. \end{array}$$

The value of Dice is between 0 and 1. Higher Dice indicates better overlap between segmented volumes and the reference standard.

*(2) Root Mean Squared Error.* The RMSE calculates the distance between the corresponding points on the automatically segmented and reference boundaries, defined by

(7) $$\begin{array}{c} \text{RMSE}={\left(\;\frac{1}{N}\sum_{i=1}^{N}{\left(\;x_{s,i}-x_{r,i}\;\right)}^{2}+{\left(\;y_{s,i}-y_{r,i}\;\right)}^{2}\;\right)}^{1/2}, \end{array}$$

where (*x*_*s*,*i*_, *y*_*s*,*i*_) is a point on the segmented boundary and (*x*_*r*,*i*_, *y*_*r*,*i*_) is the closest point to (*x*_*s*,*i*_, *y*_*s*,*i*_) on the reference boundary. The lower RMSE, the better performance.

*(3) Reliability.* The reliability function is used to assess the reliability of segmentation method, defined as

(8) Rd=Number  of  volumes  segmented  with  Dice>dTotal  number  of  volumes,

where *d* ∈ [0,1]. *ℛ*(*d*) represents the reliability in yielding Dice *d*.

*(4) Correlation Coefficient. R* between *V*_*s*_ and *V*_*r*_ is used to assess the quality of a least-squares fitting, given by

(9) R=n∑i=1nVs,iVr,i−∑i=1nVs,i∑i=1nVr,in∑i=1nVs,i2−∑i=1nVs,i21/2n∑i=1nVr,i2−∑i=1nVr,i21/2.

The value of *R* ranges from 0, no match between the two volumes, to 1, a perfect match.

## 3. Results

### 3.1. Qualitative Evaluation

**Figure 7:**
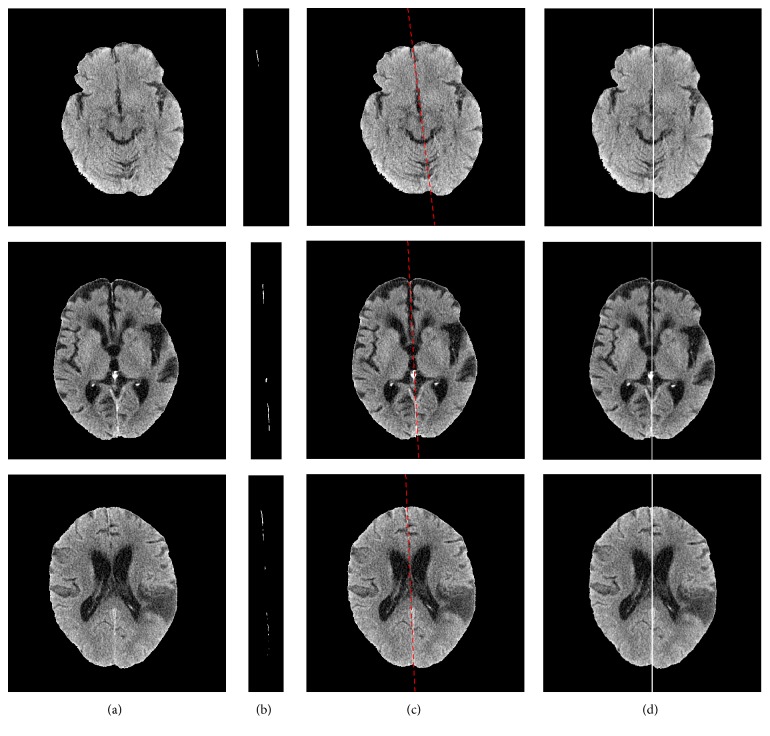
Alignment performance: original image without skull (a); detected light curve/segment (b); determined midsagittal line with red color for each slice based on 3D fitting of light curves (c); aligned brain image, where the white line shows the midline of the image (d).

Figure 7 displays the alignment of three representative brain images. The original images were shown in (a). (b) to (d) were the segmented light curve/segment, determined midsagittal line, and the final aligned result, respectively. Only a short light curve segment was detected in the brain image of the first row; however, our algorithm still accurately determined the midsagittal line, which was attributable to 3D fitting of the middle sagittal plane based on segmented light curve/segments. We can find that our alignment algorithm yielded good performance.

**Figure 8:**
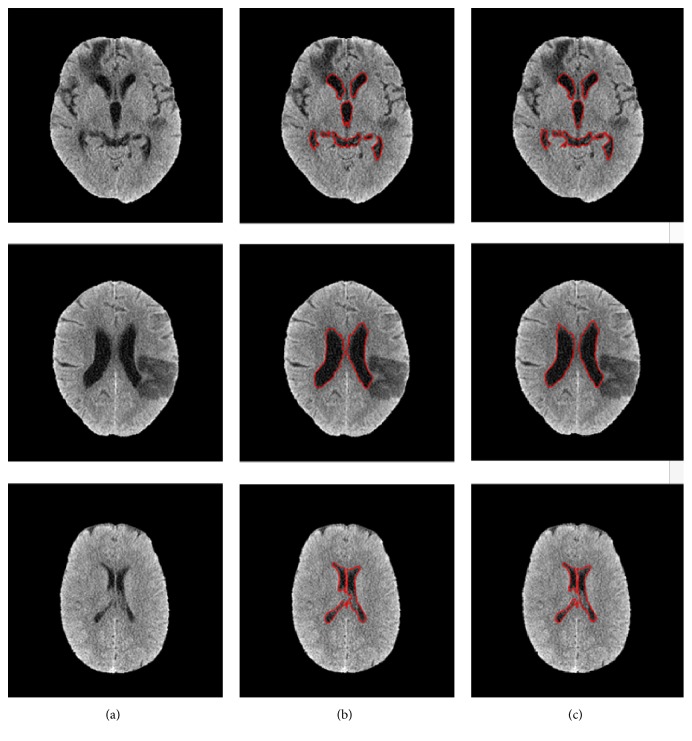
Performance of ventricle segmentation: original brain images (a); ventricle segmentation result outlined with red contours (b); contours of the reference ventricle (c).

Figure 8 shows the results of ventricle segmentation. The original brain image, ventricle segmentation result, and reference standard were shown in (a) to (c), respectively. Although some stroke regions were attached to the ventricle in original images, they were all excluded in the segmentation results. This result means that our proposed segmentation method can obtain satisfactory results on images with ischemic stroke.

### 3.2. Quantitative Evaluation Results

We quantitatively assessed the ventricle segmentation results using Dice, RMSE, the reliability (*ℛ*) and correlation coefficient (*R*). The mean Dice, sensitivity, specificity, and RMSE were 0.9447, 0.969, 0.998, and 0.219, respectively, as shown in Table 1. The analysis results of these metrics confirm the desirable performance of our proposed method.

**Figure 9:**
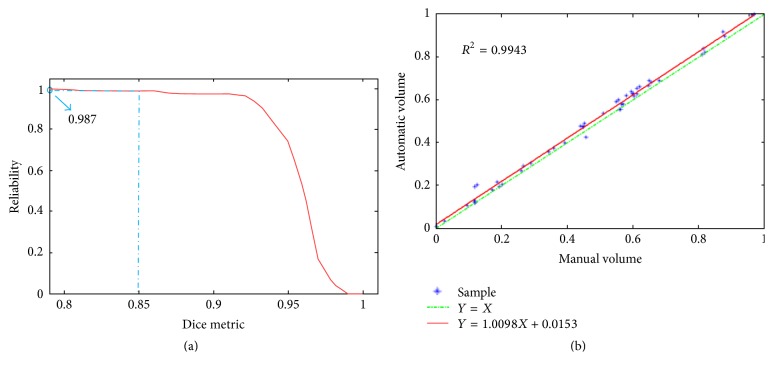
(a) The reliability of our method: *ℛ*(0.85) = 0.987; (b) segmentation volumes of our method versus manual volumes: *R* = 0.997.

The proposed method produced a reliability of *ℛ*(0.85) = 0.987 for ventricle segmentation, which means all these cases have a good agreement (Dice > 0.85).  Figure 9(a) plots *ℛ* as a function of *d*  (*d* ≥ 0.78) for the ventricle segmentation. It further shows the acceptable performance of the proposed method.

The correlation coefficients between automatic segmentation result and reference standard are 0.994. The linear regression plotted in Figure 9(b), which indicates a close correlation between the results of the proposed method and the reference standard.

## 4. Discussion

The stroke regions on CT are often adjacent or connected to the ventricle, and their intensities are similar, which makes it highly difficult for accurate segmentation of the ventricle. To achieve this goal, we developed a combined segmentation strategy composed of connectivity, image difference method, and adaptive template method that is developed to exclude stroke regions from the ventricular segmentation result, which constitutes the major strength of our segmentation scheme.

Image difference method was used to extract the large lesion regions. In this approach, the most critical step was to search the critical threshold for obtaining the ventricular segmentation result without stroke regions. This result served as “benchmark ventricular mask,” and acted as the subtrahend in the image difference method. However, the edge checking method only worked well for the large stroke regions, so this method was not able to efficiently detect the small stroke areas when they presented in the segmentation result from the critical threshold. If the benchmark ventricular mask contains small stroke areas, these small stroke regions would be left in the final segmentation results. Therefore, the adaptive template method was developed to remove these small stroke regions, which would further break up the connectivity relationship between the lesion regions and the ventricular region in 3D space. Finally, we took the largest 3D connected component in the segmentation as the ventricular region to refine the results.

The limitation of this segmentation system is that some small stroke region may still exist in the segmentation result, due to the local property of the adaptive template, which covers the main part of the ventricle. Differentiation of the ventricle and stroke region is a challenging task. In the future, we will combine the prior template of the ventricle and adaptive template to exclude the stroke region in the initial segmentation result. Besides, we will collect more data to validate our proposed segmentation system.

## 5. Conclusion

The accurate ventricle segmentation is a critical step in the development of CAD for acute ischemic stroke. Since ischemic stroke regions are generally adjacent to the brain ventricle with similar intensity, it is a challenging task to segment ventricle. In this study, we developed an objective segmentation system of brain ventricle in CT. We proposed three different schemes to exclude the stroke regions from initial segmentation, which are the main contributions in this work. The experiments illustrate the proposed segmentation method that can obtain a good performance for segmentation of ventricle in brain CT scans with ischemic stroke, which would significantly facilitate ischemic stroke region localization.

## Figures and Tables

**Table 1:** Quantitative performance evaluations (Dice, sensitivity, specificity, and RMSE) on 50 cases of patients with ischemic stroke regions.

	Mean	SD	Min	Max
Dice	0.945	0.036	0.801	0.985
Sensitivity	0.970	0.027	0.892	0.997
Specificity	0.998	0.00	0.996	0.999
RMSE (mm)	0.219	0.472	0.007	2.536
